# The Overlap Syndrome

**DOI:** 10.7759/cureus.3453

**Published:** 2018-10-15

**Authors:** Shantanu Singh, Harleen Kaur, Shivank Singh, Imran Khawaja

**Affiliations:** 1 Pulmonary Medicine, Marshall University School of Medicine, Huntington, USA; 2 Neurology, Univeristy of Missouri, Columbia, USA; 3 Internal Medicine, Southern Medical University, Guangzhou, CHN

**Keywords:** chronic obstructive pulmonary disease (copd), obstructive sleep apnea, sleep, overlap syndrome

## Abstract

The overlap syndrome (OS) was first coined by David C. Flenley in 1985 to describe the coexistence of obstructive sleep apnea (OSA) in patients with chronic obstructive pulmonary disease (COPD). Patients with OS experience more profound nocturnal oxygen desaturation (NOD) than patients with OSA or COPD alone. This underlying hypoxia in OS increases the risk of cardiovascular disease including atrial fibrillation, right heart failure, and pulmonary hypertension, thereby increasing the mortality associated with the disease. Keeping in mind the risk of mortality, it is crucial for clinicians to clinically evaluate the patients with OSA or COPD for the occurrence of OS and provide effective treatment options for the same. This review aims to highlight the pathophysiology and the risks associated with the OS along with early detection and appropriate management protocols to reduce the mortality associated with it.

## Introduction and background

The term overlap syndrome (OS) was coined by David C. Flenely in 1985 to describe the association between obstructive sleep apnea (OSA) with breathing disorders like chronic obstructive pulmonary disease (COPD) in a patient [1]. The OS is defined as the coexistence of apneas or hypopneas during sleep secondary to obstruction of the upper airway in patients with COPD. During sleep, patients with COPD experience nocturnal hypoxemia and hypoventilation mainly during the rapid eye movement (REM) phase of the sleep due to relaxation of inter-costal muscles and reduced chest wall mobility. On the other hand, patients with OSA experience episodes of apnea and hypopnea mainly through upper airway collapse, reduced intrathoracic pressures, and activation of the sympathetic nervous system resulting in nighttime arousals and excessive daytime sleepiness [2]. These episodes of nocturnal oxygen desaturation (NOD) with hypercapnia and hypoxemia are more profound in patients with OS in comparison to COPD or OSA alone. The coexistence of OS may further increase the risk of cardiovascular events particularly pulmonary hypertension and atrial fibrillation, thereby resulting in poor outcome and increased risk of mortality than in patients with COPD or OSA alone.

Understanding sleep in COPD and OSA

Patients with COPD often complain of poor quality of sleep with daytime fatigue. Sleep disorders are considered as the third most common factor affecting the quality of life in COPD patients, after dyspnea and tiredness [3]. COPD patients have difficulty in initiating or maintaining sleep with frequent nighttime arousal.

As clinicians, it is important to understand and differentiate between isolated nocturnal hypoxemia in COPD versus hypoxemia due to obstructive airway hypoventilation in OSA patients. Nearly 70% of COPD patients report isolated nocturnal hypoxemia with daytime normal saturation of 90%-95% [4]. Isolated hypoxemia is defined as the partial pressure of oxygen (PaO_2_) less than 55 mmHg or oxygen saturation of less than 88% for at least five minutes during sleep [5]. Sleep hypoventilation is defined as the increase in partial pressure of carbon dioxide (PaCO_2_) /hypercapnia more than 55 mmHg for 10 minutes during sleep or increase in PaCO_2_ by 10mmHg above awake supine value over 50mmHg for 10 minutes [6].

The physiological derangements seen during sleep in OS include increased upper airway resistance, ineffective gas exchange, and impact of central sleep drive. Sleep is affected generally in patients with COPD because of its impact on respiratory control, respiratory muscle function and lung mechanics [7].

During sleep, the base of the tongue and soft palate are the two main regions responsible for increased airflow resistance. In the supine position, these structures result in anterior-posterior narrowing at the level of the pharyngeal wall, leading to increased upper airway resistance. Further, during inspiration, the negative intraluminal pressure results in lateral pharyngeal wall intrusion leading to circumferential airway collapse. This oropharyngeal crowding can be more pronounced in obese individuals resulting in OSA. Further with this increased upper airway resistance, the diaphragmatic and abdominal muscle have to exert more effort to maintain airflow to lower respiratory tract during sleep. Also, in COPD patients, the cough reflex is reduced during sleep resulting in mucus plugging (due to the ineffective clearing of airway secretions) which further reduces the alveolar ventilation.

The disordered breathing in COPD patients is mainly seen in the REM phase of sleep due to reduced intercostal muscle activity and chest wall mobility [8]. This hypotonic muscle activity during sleep results in a severe drop in minute ventilation in COPD patients. It is estimated that the fall in minute ventilation from wakefulness to non-REM sleep is approximately 16% and the minute ventilation drops further to 32% in REM sleep [9]. This drop-in minute ventilation and the decrease in tidal volume result in more pronounced hypoxia and hypoventilation. Further reduced chest wall mobility and relaxation of intercostal muscles during REM phase precipitates nocturnal hypoxia and reduces the functional residual capacity (FRC) in these patients. This results in rapid shallow breathing pattern during the REM sleep which further reduces the alveolar ventilation. Last of all, during sleep, the central respiratory drive is blunted due to profound hypoxia and hypercapnia in patients with COPD and OS.

## Review

Epidemiology

COPD is the third most common cause of death in the United States accounting for 4% of all the deaths [10]. The Burden of Obstructive Lung Disease (BOLD) study estimates 19% prevalence of COPD in the middle-aged western population [11]. According to the National Health and Nutrition Examination Survey III (NHANES), the prevalence of COPD ranges from 16.8% for Global Initiative for Obstructive Lung Disease (GOLD) stage I (mild, (forced expiratory volume) FEV_1_ >80%) and 7.7% for GOLD stage II (moderate, FEV_1_ range 50%-79%) [12]. However, another study showed that patients with severe COPD of GOLD stage III (severe, FEV_1_ range 30% to 49%) and IV (very severe, FEV_1_ <30%) had lower body mass index (BMI) from the systemic effects of the disease which resulted in fewer episodes of apnea-hypopnea index (AHI) thereby reducing the prevalence of OSA in these patients [13]. Further, the prevalence of OSA alone in the United States is approximately 30% [14]. A recent review by Shawon et al. concluded that the prevalence of OS ranges from 2.9% to 65.9% in patients with COPD [15]. However, there are some conflicting reports suggesting no significant prevalence of OSA in COPD. The data from Sleep Heart Health Study suggests that mild obstructive lung disease did not have an association with OSA [16]. Further, the mild airflow obstruction did not affect the respiratory disturbance index (RDI) and Epworth sleepiness score (ESS) in these patients. The major limitation of this study was that most patients suffered only from mild airflow obstruction. Likewise, the Multinational Monitoring of Trends and Determinants in Cardiovascular Disease (MONICA) II study also concluded that no higher prevalence was noted between two disorders [17]. The study population included 663 subjects with COPD, in which OS was noted in 1% of the patients.

Pathophysiology

NOD is considered as the most common consequences of disturbed sleep in COPD. The three mechanisms that contribute to NOD in COPD involve alveolar hypoventilation, decreased ventilation-perfusion matching, and decreased end-expiratory volume (ERV). Patients with OS have increased risk of NOD as compared to COPD patients resulting in respiratory failure and hypercapnia.

The underlying hypoxia in these patients induces the release of systemic inflammatory mediators including C-reactive protein (CRP), interleukin-6 (IL-6), nuclear factor-kappa beta (NF-ĸβ), tumor necrosis factor-alpha (TNF-α) and interleukin-8 (IL-8) [18-20]. This intermittent hypoxia also induces oxidative stress that results in the release of reactive oxygen species (ROS) mainly leukocytes [21]. The release of systemic inflammatory mediators further result in endothelial dysfunction and enhanced atherosclerosis.

Cigarette smoking is a common risk factor for both COPD and OSA. Smoking can also promote oxidative stress and release of inflammatory mediators thereby accelerating the underlying pathophysiologic process [22]. Obesity is considered as the key risk factor for OSA. Neck obesity results in upper airway narrowing predisposing to NOD in patients with COPD and OSA. Truncal obesity may reduce the chest wall compliance and respiratory muscle strength thereby resulting in ventilatory disturbances and ventilation-perfusion mismatching [23]. Patients with advanced COPD are likely to have low BMI and diminished REM sleep that may be protective for the development of OSA. Certain medications like theophylline, inhaled anticholinergics, and beta-agonist may help to improve the NOD by reducing the gas trapping and lower airway obstruction [24-25]. On the other hand, the use of corticosteroids may result in central obesity and fluid restriction thereby promoting upper airway narrowing.

Clinical consequences of OS

Patients with OS have increased risk of mortality due to cardiovascular events [26]. Hypoxic drive results in oxidative stress and stimulates the release of systemic inflammatory mediators like TNF-α, IL-6, IL-8, CRP which ultimately results in endothelial dysfunction and atherosclerotic plaque formation [27]. OSA also results in metabolic dysfunction including insulin resistance and abnormal lipid metabolism. OSA is also associated with systemic hypertension which increases the risk of coronary artery disease, congestive heart failure, arrhythmias and stroke [28]. The Wisconsin Sleep Cohort study provided data highlighting a greater risk of cardiovascular mortality in patient with OSA, and improvement in morbidity and mortality in OSA patients with the use of CPAP therapy [29].

**Figure 1:**
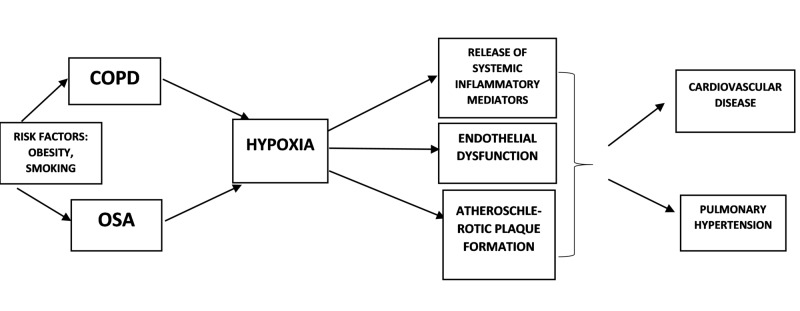
Summary of clinical consequences of overlap syndrome COPD: chronic obstructive pulmonary disease; OSA: obstructive sleep apnea

Patients with OS have increased risk of pulmonary hypertension and right heart failure secondary to underlying NOD, daytime hypoxemia and hypercapnia as compared to patients with COPD and OSA alone. Hawrylkiewicz et al. observed in their study that 86% subjects with OS had pulmonary hypertension as compared to 16% subject with OSA alone [30]. Chaouat and coworkers observed pulmonary hypertension in 36% of patients with OS as compared to 7% of patients with COPD [31]. The increased risk of developing pulmonary heart disease has also increased the frequency of deaths observed in sleep in OS syndrome [32]. There is also increased risk of rhythm disorders noted in OS. There is a significant risk of premature ventricular contractions and nocturnal desaturations in COPD patients and increased risk of atrial fibrillation in OSA. A retrospective cohort study involving 2,873 patients showed increased evidence of new-onset atrial fibrillation in patients with OS as compared to COPD or OSA alone [33]. The following illustration summarizes the clinical consequences of OS (Figure 1).

Clinical assessment

It is crucial for clinicians to evaluate patients with OSA or COPD for OS. Patients with COPD will generally report poor sleep quality and daytime fatigue while patients with OSA generally present with excessive snoring, unrefreshed sleep, and excessive daytime sleepiness.

Polysomnography is the gold standard to detect sleep disorders in OS. However overnight oximetry can be used as a simple screening test to detect nocturnal hypoxia during sleep. A cyclical (sawtooth) pattern on overnight oximetry may indicate sleep apnea in COPD patients and should be confirmed by polysomnography studies [34].

The Sleep Heart Health study suggested that patients with mild COPD (GOLD stage I and II) may not reflect classic symptoms of OSA or may not have disturbed quality of sleep. In proper clinical settings, they should be evaluated with polysomnography to rule out sleep apnea [35]. Further, studies by Chaouat and coworkers also emphasize that patients with OS with underlying pulmonary hypertension may only reflect mild abnormality on spirometry and oxygenation studies [36]. Also, guidelines from the American Thoracic Society/European Respiratory suggest overnight testing in patients with mild COPD with underlying pulmonary hypertension [37].

Treatment

OS is associated with an increased risk of morbidity and mortality than each of the disease alone. Treatment of OSA in COPD patients can reduce cardiovascular mortality and improve the survival rate in these patients [38]. Mermigkis et al. noted in their study that based on St. George’s Respiratory Questionnaire, patients with OS have a significantly worse quality of life as compared to COPD patients alone [39]. The treatment of OS should aim to maintain nocturnal oxygenation and reduce the episodes of hypoxemia and hypoventilation along with improving the sleep quality.

1. Lifestyle Modifications

Structured exercise program and pulmonary rehabilitation are shown to be beneficial in both OSA and COPD. Structured exercise programs aim to improve the skeletal muscle wasting in patients with COPD. Likewise, structured exercise program in OSA patients have shown improvement in AHI, daytime sleepiness, and overall sleep quality [40] However structured exercise program alone may not be effective in the management of the disease and should be supplemented with appropriate treatment options. Patients with COPD have reported improvement in the quality of life, mood index, dyspnea scores, and reduced hospital frequency with pulmonary rehabilitation. The effect of pulmonary rehabilitation was further seen by Solar et al. in 64 patients with COPD. According to the Pittsburg Sleep Quality Index (PSQI), initially 58% of subjects reported poor sleep quality, and by the end of the eight-week period, the number reduced to 19% [41]. Along with these behavioral modification and exercise program, cessation of smoking is highly advisable to reduce the morbidity and mortality associated with the disease.

2. Supplemental Oxygen Therapy

It is the mainstay of management of COPD. Studies have shown that supplemental oxygen therapy for more than 18 hours a day including during sleep can help to improve daytime and nocturnal hypoxemia and reduce the risk of mortality in these patients. However, oxygen therapy may not be effective in the management of OSA. It can help to reduce the episodes of NOD but does not improve the quality of sleep or nighttime arousals [42]. Alford et al. performed a study by administering four liters of supplemental oxygen to 20 men with OS and noted improvement in NOD but with increased frequency of obstructive events associated with hypercapnia and decrease in pH [43]. Hence supplemental oxygen therapy is not recommended as a definitive treatment option for OS.

3. Bronchodilators and Corticosteroids

Treatment of COPD can improve the NOD and reduce the need for supplemental oxygen therapy. Martin et al. noted improvement in NOD, sleep quality, and increase in REM sleep time in patients with moderate to severe COPD (FEV_1_< 65%) treated with inhaled ipratropium four times a day [44]. Similar benefits were noted with long-acting beta-agonist and oral corticosteroid therapy in COPD patients [45-46]. However, less is known if the treatment of COPD in OS can ameliorate symptoms of OSA.

4. Continuous Positive Airway Pressure (CPAP)

CPAP therapy is the most effective treatment for OSA and OS. It reduces the upper airway resistance thereby reducing the nocturnal hypoventilation. It is also effective in limiting the release of ROS in OSA. CPAP therapy is effective in improving the FEV_1_, PaO_2_, PaCO_2_ and mean pulmonary artery pressure [47]. Marin and his colleagues concluded in their study that treatment of OSA can improve the mortality in patients with OS. Also, CPAP therapy can improve the survival outcomes in patients with COPD and OSA and is also effective in reducing COPD exacerbations in OS. Nocturnal CPAP therapy in OS has also shown to improve the walking capacity and exercise tolerance in these patients [48].

5. Non-invasive Ventilation (NIV)

NIV is effective in the management of COPD exacerbations mainly in hypercapnic respiratory failure. NIV has shown to improve nocturnal hypoxemia and quality of sleep in COPD [49]. It also helps to improve respiratory muscle strength and endurance in these patients. Long-term use of NIV in COPD patients helps to improve lung compliance, prevents atelectasis, and reduce the work of breathing. While the use of NIV is well established in COPD, limited literature is available to understand its benefit in OS or OSA.

## Conclusions

Keeping in mind the increased risk of cardiovascular morbidity and mortality with OS, it is important for clinicians to screen COPD patients with OSA and vice versa. Patients with a high index of suspicion should be clinically assessed and advised effective treatment options for the same. The two clinical disorders should be treated simultaneously to achieve optimal nocturnal oxygen saturation, prevent multiple arousals, and improve quality of life, which may help to reduce the morbidity and mortality associated with OS.
